# Oxygen impairs oligodendroglial development via oxidative stress and reduced expression of HIF-1α

**DOI:** 10.1038/srep43000

**Published:** 2017-02-23

**Authors:** Christina Brill, Till Scheuer, Christoph Bührer, Stefanie Endesfelder, Thomas Schmitz

**Affiliations:** 1 Department of Neonatology, Charité University Medical Center, Berlin, Germany; 2 Institute of Bioanalytics, University of Applied Sciences, Berlin, Germany

## Abstract

The premature increase of oxygen tension may contribute to oligodendroglial precursor cell (OPC) damage in preterm infants. Fetal OPCs are exposed to low oxygen tissue tensions not matched when cells are cultured in room air. Maturation (A2B5, O4, O1, MBP, CNP, arborization), oxidative stress (nitrotyrosine Western blot, NRF2 and SOD2 expression), apoptosis (TUNEL), proliferation (Ki67), and expression of transcription factors regulated by Hypoxia-Inducible-Factor-1-alpha (Hif-1α) expressed in OPCs (Olig1, Olig2, Sox9, Sox10) were assessed in rat OPCs and OLN93 cells cultured at 5% O2 and 21% O2. Influences of Hif-1α were investigated by Hif-1α luciferase reporter assays and Hif-1α-knockdown experiments. At 21% O2, cell proliferation was decreased and process arborization of OPCs was reduced. Expression of MBP, CNP, Olig1, Sox9 and Sox10 was lower at 21% O2, while Nrf2, SOD2, nitrotyrosine were increased. Apoptosis was unchanged. Luciferease reporter assay in OLN93 cells indicated increased Hif-1α activity at 5% O2. In OLN93 cells at 5% O2, Hif-1α knockdown decreased the expression of MBP and CNP, similar to that observed at 21% O2. These data indicate that culturing OPCs at 21% O2 negatively affects development and maturation. Both enhanced oxidative stress and reduced expression of Hif-1α-regulated genes contribute to these hyperoxia-induced changes.

In addition to its fundamental role in energy metabolism, oxygen serves as a regulator of cellular development. Cells of the central nervous system, in particular, are known to be highly susceptible to varying oxygen tensions^1^. While during fetal brain development, the *in utero* environment composes low arterial oxygen levels of 20–25 mmHg^2^, birth into room air causes a several fold increase of oxygen in the infant and in its brain. In preterm infants, however, this rise of oxygen occurs prematurely and may interfere with crucial cellular processes during early brain development.

In neural precursor cells, for example, higher *in vitro* oxygen levels (20%) induce apoptotic cell death while low oxygen (e.g. 5%) promotes the expansion of clonal precursor populations^3^. In astrocytes, variant oxygen tensions *in vitro* have been shown to result in different transcription patterns for ribosomal activity, immune responses, and cell cycle regulation^4^ and lower oxygen levels of 7% during reoxygenation were found to reduce cell death in astroglia after oxygen-glucose-deprivation^5^. In fact, the 21% O_2_ commonly used for cell cultures produce fairly high partial oxygen pressures of more than 150 mmHg, whereas under physiological conditions in the cerebral cortex, neural cells are exposed to much lower oxygen tensions of about 16–38 mmHg (2–5% O_2_)^6^,^7^.

These circumstances, however, have not yet been investigated with regards to the development of oligodendroglia. While the developmental process itself has been well described^8^,^9^,^10^, oligodendroglial precursor cells (OPCs) are known to have pronounced susceptibility to oxidative stress and to radicals due to their low levels of anti-oxidants and radical scavangers^11^,^12^. In primary cultured OPCs, oxidative stress caused by exposure to peroxides disrupts oligodendroglial maturation and downregulates gene expression of factors needed for oligodendroglial development^13^. Perturbation of immature neural cell development by high oxygen could thus be mediated by oxygen-induced oxidative stress. Alternatively, decreased hypoxia-inducible-factor-1-alpha (HIF-1α) may also disrupt cellular development.

Under hypoxic conditions, HIF-1α is stabilized and serves as a key transcriptional factor for various regulatory pathways^14^. High HIF-1α expression under hypoxia also coincides with increased activity of sonic hedgehog in the rat embryo heart^15^ and sonic hedgehog is known to promote the expansion of the oligodendroglial population during development and after injury of the CNS^16^,^17^,^18^. Several genes relevant to cell survival have been found to be upregulated by hypoxia via the HIF-1α pathway^19^ and neuroprotective pre-conditioning prior to an injurious challenge by hypoxia-ischemia has just recently been demonstrated to depend on the presence of HIF-1α^20^.

Hence, we hypothesize that survival, proliferation and maturation of immature oligodendroglial lineage cells may be affected by the level of environmental oxygen. In our *in vitro* studies, we therefore investigated whether HIF-1α and/or oxidative stress are involved in specific changes of oligodendroglial development in response to oxygen. The results may help to understand the mechanisms of oligodendroglial damage in the brains of preterm infants potentially caused by the drastic increase of oxygen levels after birth.

## Results

### Lower oligodendroglial cell numbers at higher oxygen levels

**Figure 1:**
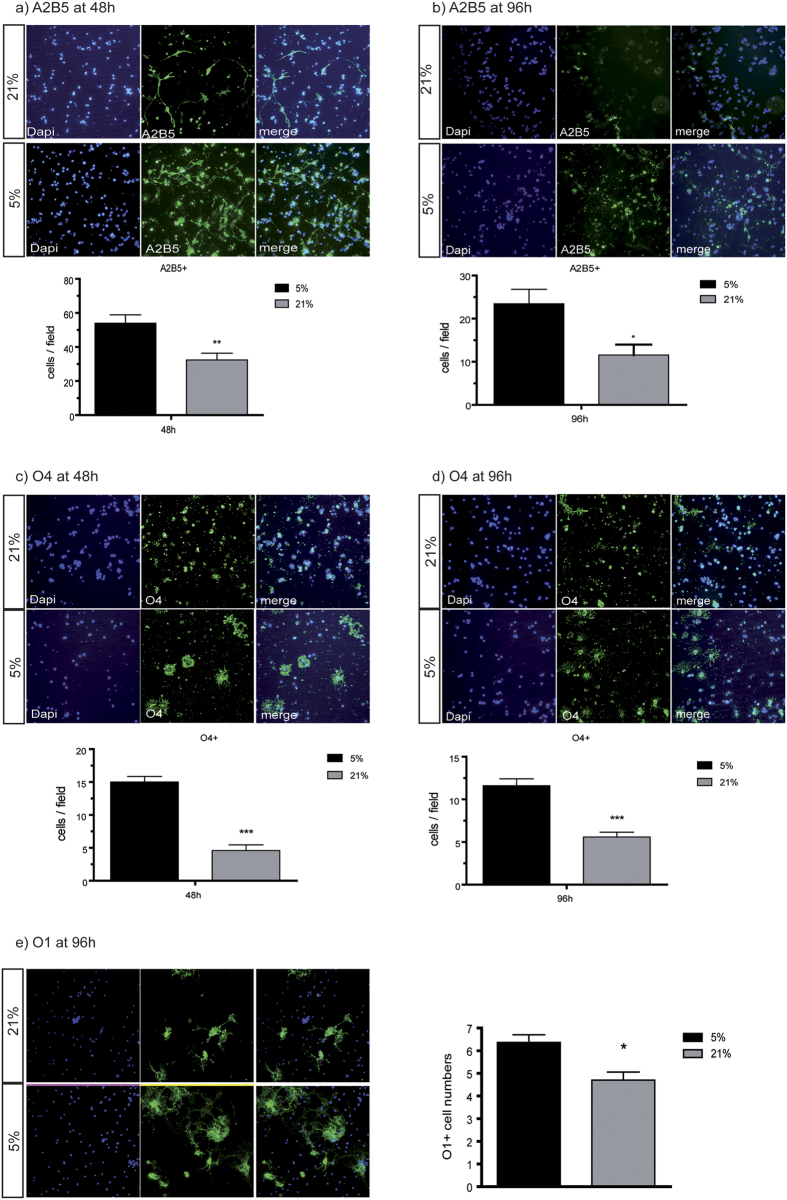
Decreased numbers of OPCs cultured at 21% O_2_. Immunocytochemistry of OPC shows a significant reduction in cell numbers of A2B5+ OPCs cultured in 21% O_2_ compared to 5% O_2_ after 48 hours (**a**) p = 0.0029) and 96 hours (**b**) p = 0.024) culture time and a highly significant reduction of O4+OPC cultured in 21% O_2_ after 48 hours (**c**) p < 0.0001) and 96 hours (**d**) p < 0.0001) culture time. (N = 15, unpaired t-test with Welch’s correction *p < 0.05, **p < 0.01, ***p < 0.001). Numbers of O1 positive cells (**e**) are reduced after 96 h culture time at 21% compared to 5% (N = 6, *p < 0.05).

In order to analyze whether oxygen tensions influence oligodendroglial development we quantified total numbers of primary rat oligodendroglial lineage cells that were cultured for 48 and 96 hours in either 5% or 21% O_2_. We used immunocytochemistry with different oligodendroglial markers to characterize different stages of maturation. Oligodendroglial precursor cells (OPC) were labeled using antibodies against A2B5 antigen and immature oligodendroglia were marked using anti-O4 antibodies. The results show that A2B5+ cells were decreased in number in 21% oxygen when compared with 5% O_2_. The reduction of OPCs at 21% O_2_ was found after 48 hours as well as after 96 hours culture time (at 48 hours: 53.98 cells/field at 5% O_2_ vs. 32.39 cells/field at 21% O_2_, p = 0.0029; at 96 hours: 23.44 cells/field at 5% O_2_ vs.11.58 cells/field at 21% O_2_, p = 0.024) (Fig. 1a,b) A pronounced decrease in the numbers of immature O4+ oligodendroglia was observed after 48 and 96 hours (at 48 hours: 15.99 O4+ cells/field at 5% O_2_ vs. 4.60 O4+ cells/field at 21% O_2_, p < 0.0001, at 96 hours: 11.59 O4+ cells/field at 5% O_2_ vs.5.60 cells/field at 21% O_2_, p < 0.0001) (Fig. 1c,d). Similar results were found for O1+ cells after 96 hours culture time (5% O_2_ = 6.36+/−0.69 [SEM] cells per field vs. 21% O_2_ = 4.70+/−0.71 cells per field; N = 8) (Fig. 1e). This loss of cells expressing A2B5, O4 and O1 at 21% O_2_ could be due to increased cell death, impaired antigen expression, or decreased proliferation.

### Lower oxygen levels lead to enhanced oligodendroglial proliferation *in vitro*

During oligodendrocyte development, the expansion of the oligodendroglial lineage population proceeds first by progenitor proliferation and is followed by maturation and functional refinement^8^,^21^,^22^. We first determined the influence of the different oxygen levels on oligodendroglial proliferation *in vitro*. We used immunocytochemistry for Ki67 as a widely established marker for proliferating cells in the S- and G2-phase, together with antibodies against A2B5 to identify OPCs and against O4 to analyze immature oligodendroglia.

**Figure 2:**
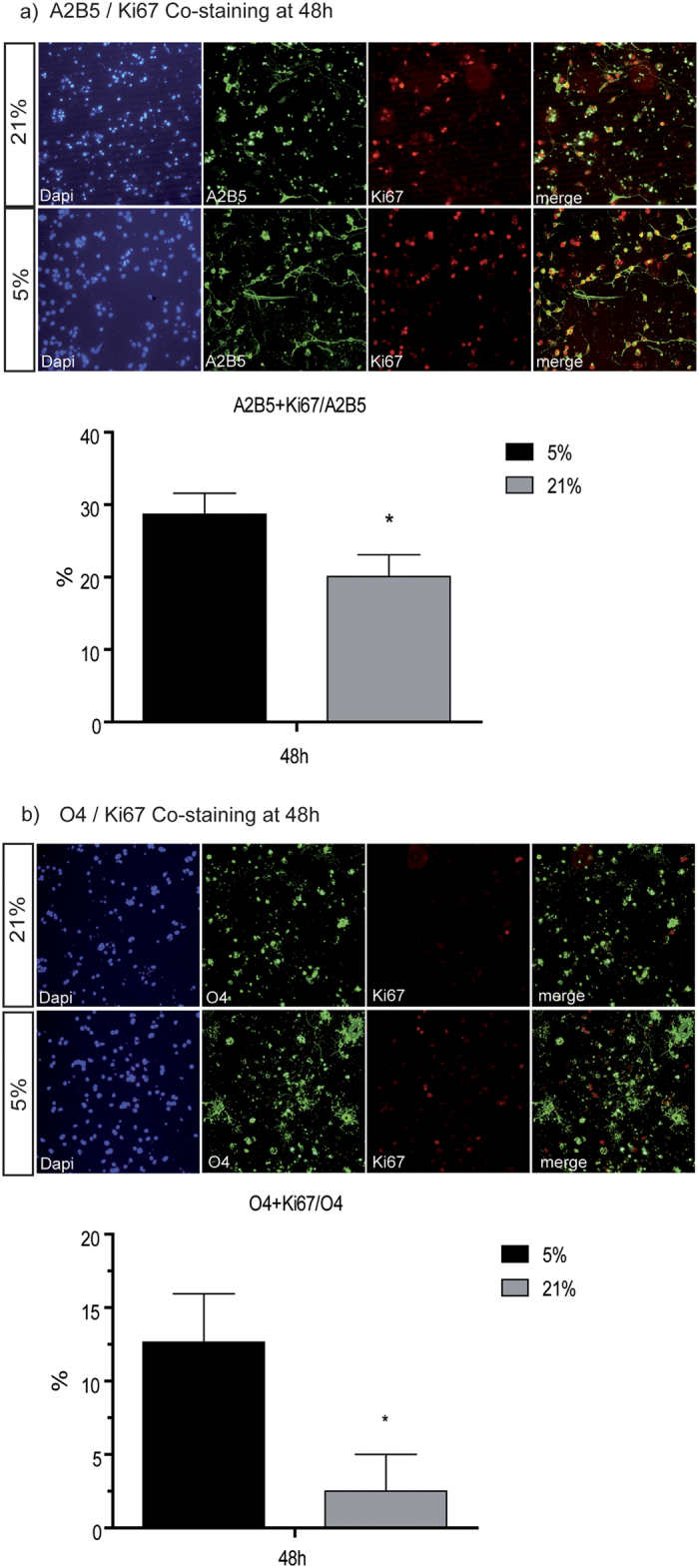
Proliferation of OPC and O4+ oligodendroglia is affected by oxygen levels. Co-staining of OPC for oligodendroglial markers and proliferation marker Ki67 shows a significantly decreased percentage of A2B5+OPC (**a**) p = 0.042) and O4+OPC (**b**) p = 0.022) proliferating in 21% O_2_ compared to 5% O_2_ after 48 hours (N = 15, unpaired t-test with Welch’s correction *p < 0.05).

Culture conditions at 21% oxygen for 48 hours yielded significantly lower numbers of A2B5+Ki67+ proliferating OPCs, i.e. at 5% O_2_ 16.93% of A2B5+ were also Ki67+ compared to 10.8% in cells cultured at 21% O_2_) (p = 0.042) (Fig. 2a). The quantification of O4+Ki67+ cells after 48 hours culture time revealed a strong inhibitory effect of higher oxygen on proliferation of immature oligodendroglia (12.65% of O4+ cells were also Ki67+ when cultured in 5% O_2_in comparison to only 2.5% of O4+ cells cultured at 21% O_2_, p = 0.022) (Fig. 2b).

**Figure 3:**
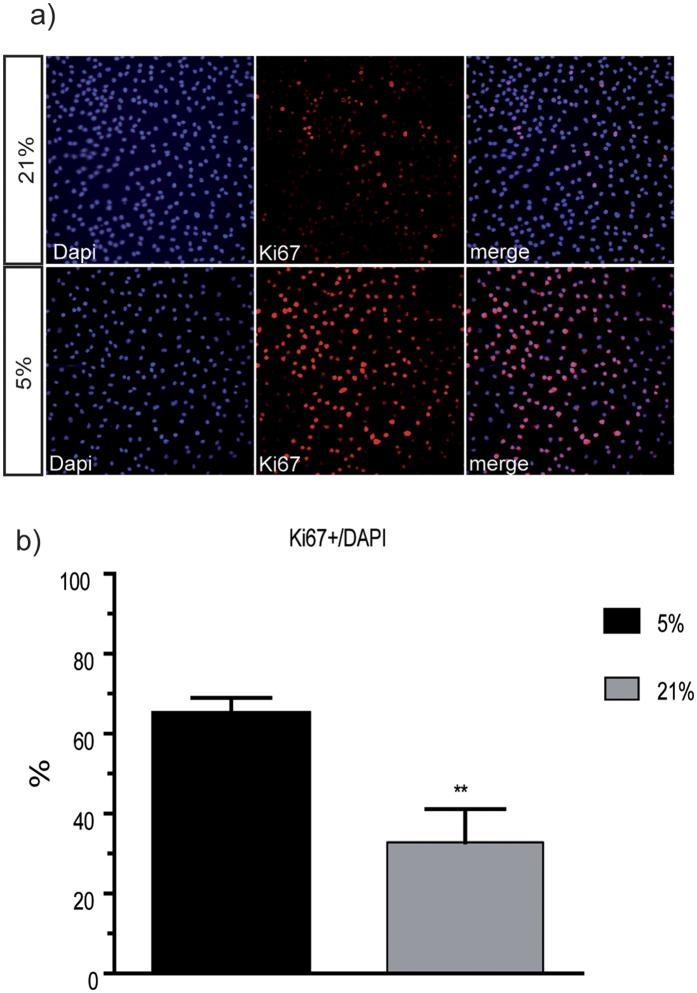
Proliferation in cultured OLN-93 cell line. Immunocytochemistry of OLN-93 cell line for proliferation marker Ki67 (**a**) shows a drastic reduction of proliferating OLN-93 cells in the 21% O_2_ culture group compared to the 5% O_2_ culture group after 48 hours culture time (**b**) (N = 5, unpaired t-test, p = 0.008; *p < 0.05, ** < p0.01).

We also used the OLN93 cell line to analyze proliferation. Though the OLN93 cell line does not express A2B5 antigen^23^ and therefore cannot be labeled with A2B5 antibodies, it resembles bipolar O-2A-progenitor cells^23^. These cells were stained with DAPI and Ki67 to mark proliferating cells after 48 hours culture time. As a result, proliferation of all OLN93 cells cultured in 21% oxygen was significantly reduced compared to 5% oxygen (Ki67+ Dapi+ cells: 65.20% of Dapi+ at 5% O2 compared to 32.8% in OLN93 cultured at 21% p = 0.008) (Fig. 3).

These results clearly indicate that decreased proliferation of oligodendroglial cells represents a mechanism of cell number reduction caused by higher oxygen levels.

### Developmental and maturational genes are downregulated by higher oxygen

To analyze the impact of 21% oxygen on oligodendroglial development more specifically, we used real-time PCR to quantify the expression of genes known to be important for oligodendroglial lineage progression (Olig1, Olig2), development (Sox9, Sox10) and for the function of mature oligodendrocytes (CNP, MBP). RNA was extracted from OPCs cultured at 21% and 5% O_2_ for 48 hours.

**Figure 4:**
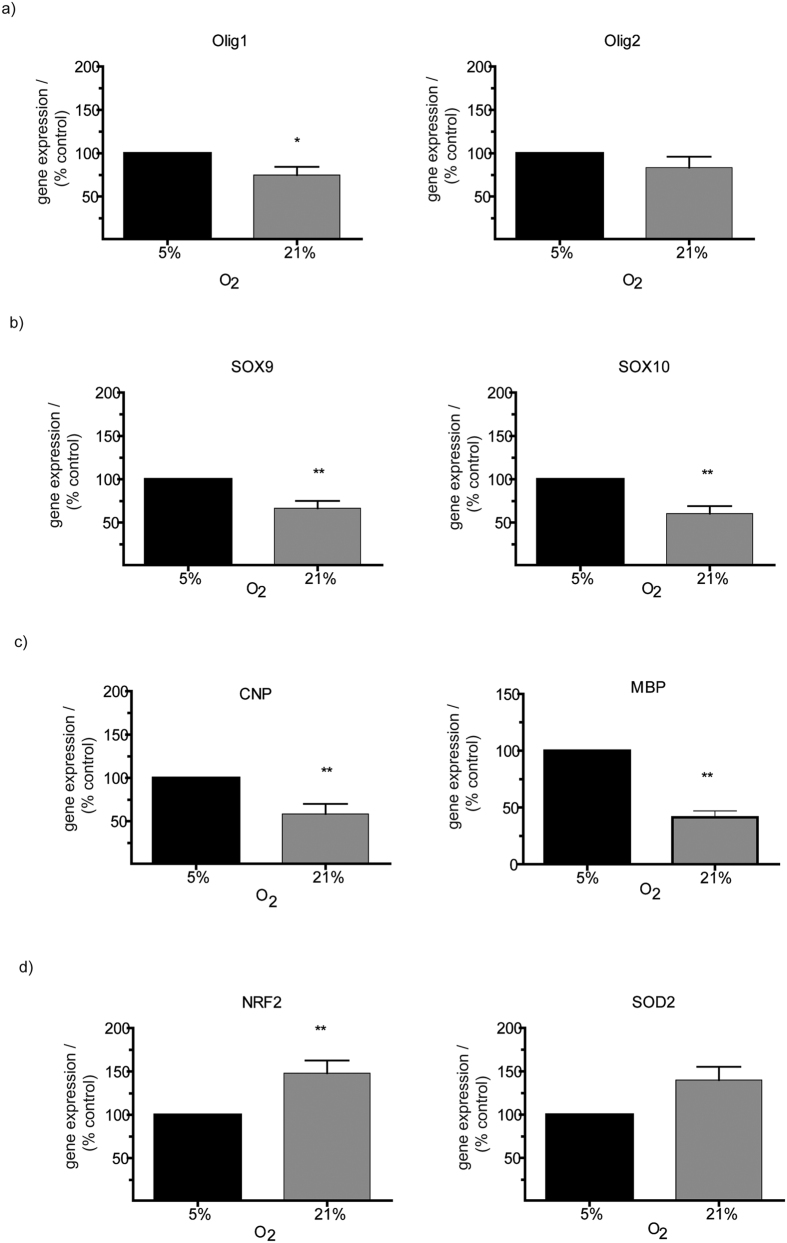
Gene expression of markers of oligodendroglial development and oxidative stress response. Realtime PCR used to quantify genexpression after 48 hours culture in 5% and 21% O_2_. Olig1 was significantly downregulated at 21% compared to 5% O_2_ (p = 0.037), Olig2 downregulation was notable. (**a**) Markers for oligodendroglial development (Sox9, p = 0.030; Sox10, p = 0.039) (**b**) and maturational markers (CNP, p = 0.0098; MBP, p = 0.002) (**c**) were drastically reduced in 21% compared to 5% O_2_. Oxidative stress response was visualized through significantly increased expression of Nrf2 (p = 0.0098) but only tended to enhance SOD2 expression (**d**) (N = 10, Wilcoxon signed rank test *p < 0.05, **p < 0.01, ***p < 0.001).

Interestingly, Olig1 was significantly downregulated by 25.29% in OPCs cultured at 21% as compared to OPCs kept in 5% O_2_ (p = 0.037) (Fig. 4a). A decline of Olig2 expression by 16.73% was also seen in OPCs at 21% but numbers were not statistically significant on statistical analysis (Fig. 4a). Remarkably, changes were more pronounced in markers of maturation and development: Sox9 and Sox10 were both drastically downregulated in OPCs cultured at 21% compared to 5% O_2_ (Sox 9: 66.52% of control, p = 0.030; Sox10: 60.36% of control, p = 0.039) (Fig. 4b). Sox9, in particular, is reported to be regulated by Hypoxia-Inducible-Factor, which suggests a possible mechanism of HIF-dependent response to changes in oxygen levels^24^. Furthermore, gene expression of CNP and of MBP was also markedly diminished in OPC cultures kept in 21% O_2_ down to 58.43%, (p = 0.0098) and to 41.42% of control levels (p = 0.002), respectively, as compared to OPC cultures kept at 5% O_2_ (Fig. 4c). Hence, the exposure to higher oxygen caused a significant downregulation of transcription factors and maturational markers in OPCs, indicating a central role of oxygen environment in the regulation of oligodendroglial development.

### Oxidative stress and apoptosis in OPCs at 21% and at 5% O_2_

Higher oxygen levels may also induce cellular stress through reactive oxygen species and/or radicals. To assess whether cultured OPCs kept in 21% O_2_ are challenged by higher oxidative stress, we analyzed expression levels of factors that are needed for cellular anti-oxidant defense, i.e*. superoxide dismutase 2* (SOD2) and *nuclear factor erythoid 2-related factor 2* (Nrf2), which in particular represents a transcription factor that orchestrates a large set of cellular anti-oxidant enzymes. The results of realtime PCR analysis showed that OPCs kept at 21% O_2_ tended to have increased SOD2 expression, but numbers were not significant on statistical analysis (139.94% of controls; p = 0.0840). In contrast, Nrf2 was significantly upregulated in OPCs cultured at 21% O_2_ (147.87% of controls, p = 0.0098). This amplified expression underlines a pronounced anti-oxidative cellular response to oxidative stimulation (Fig. 4d).

**Figure 5:**
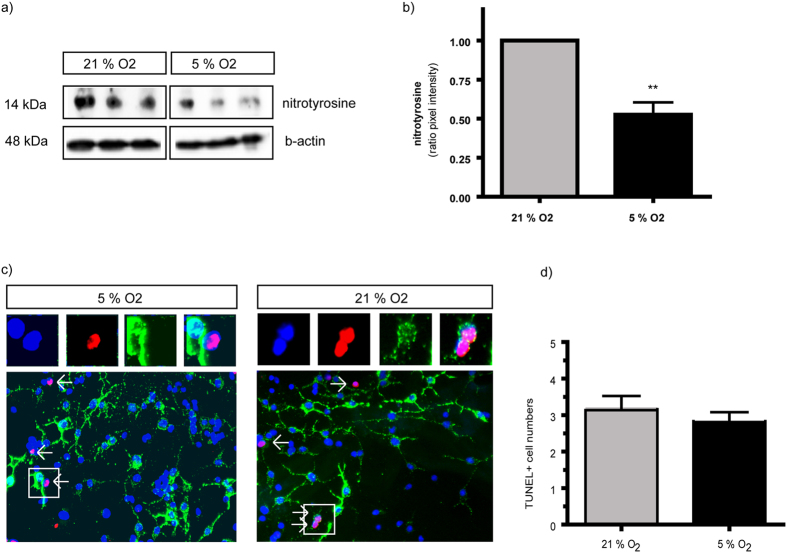
Nitrotyrosine production and TUNEL in OPC cultures at 21% and 5% O_2_ *in vitro*. Western blot analysis of nitrotyrosine intensity in protein lysates obtained from OPC cultures at 5% O_2_ compared to those at 21% O_2_ (**a**). Statistical analysis using paired ttest to directly compare the two culture conditions after 48 hours incubation time revealed a significant increase of nitrotyrosine in proteins obtained from OPCs at the higher oxygen level. (**b**) (N = 5, paired ttest *P = 0.013). Immunocytochemistry for TUNEL (red) and for A2B5 (green) in OPC cultures do not show differences in the numbers of TUNEL+ apoptotic cells at 21% O_2_ as compared to 5% O_2_ (dapi = blue) (**c,d**). The majority of dying TUNEL+ cells does not reveal co-expression of A2B5.

In order to further determine oxidative stress responses in OPC cultures we used Western blot analysis of nitrotyrosine levels, which is commonly used as a biochemical marker of nitration. As a result, nitrotyrosine levels in protein lysates obtained from OPC cultures were significantly increased after incubation at the higher oxygen culture condition with 21% O_2_ as compared to the lower 5% O_2_ (Fig. 5a,b).

To measure a potential impact of oxidative stress on apoptotic death, we performed TUNEL stainings in the OPC cultures together with A2B5 immunocytochemistry. However, the number of TUNEL+ cells was not increased in cell cultures kept in 21% O_2_ (3.18+/−0.54 cells per field) as compared to those at 5% O_2_ (2.84+/−0.41 cells per field; N = 4) (Fig. 5c,d). At both culture conditions, the vast majority of TUNEL+ nuclei were without co-localization for A2B5.

### Morphological changes in O4+ oligodendroglia

**Figure 6:**
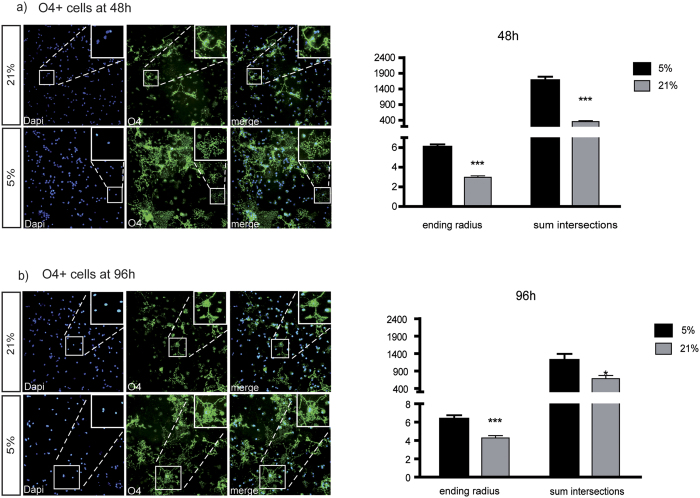
Morphological changes in primary oligodendroglia. Analysis of morphology using the ImageJ PlugIn Sholl analysis resulted in a highly significant decrease in cell size and arborization of primary O4+ immature oligodendroglia cultured at 21% O_2_ compared to 5% O_2_ after 48 hours (**a**) p < 0.0001 for both) and 96 hours (**b**) ending radius: p = 0.0009, sum of intersections: p = 0.0237) (N = 8, analyzing 4 sections in 5 plates/N, unpaired t-test with Welch’s correction *p < 0.05, **p < 0.01, ***p < 0.001).

We used Sholl analysis to designate morphological differences of OPCs cultured at 5% vs. 21% oxygen. The number of intersections of cell appendices with the concentric circles was used as a parameter of morphological complexity and the ending radius was used as a representation of cell size. O4+ cells cultured for 48 hours showed a drastic difference in cell size depending on the amount of environmental oxygen used in the cultures. The ending radius of cells cultured at 21% O_2_ only amounted to 49% of that of OPCs cultured at 5% O_2_ (after 48 hours culture time: 2.96 vs. 6.03 arbitrary units, p < 0.0001) (Fig. 6a). The assessment of morphological features in terms of sum of intersections of OPC branches with concentric circles produced by the software-plugin showed similar differences: The numbers differed from a mean of 1.663.066 intersections in cells cultured at 5% to 356.360 intersections in O4+ cells cultured for 48 hours at 21% O_2_ (p < 0.0001) (Fig. 6a), hence demonstrating a much higher morphological complexity of OPCs cultured at 5% O_2_. Sholl analysis of O4+ cells cultured for 96 hours showed similar results. Again, the mean ending radius of cells at 5% significantly exceeded that of cells cultured in 21% O_2_ (6.33 vs. 4.27 arbitrary units, respectively, p = 0.0009) (Fig. 5b), and the arborization analysis revealed a significantly more complex structure as indicated by higher numbers of intersections (1.206.300 vs. 680.938, p < 0.0237) (Fig. 6b).

Taken together, 21% oxygen consistently and over different exposure times, impaired the morphological development of O4+ immature oligodendroglia in our *in vitro* experiments.

### HIF-1α activity is reduced in oligodendroglial cells at 21% O_2_

**Figure 7:**
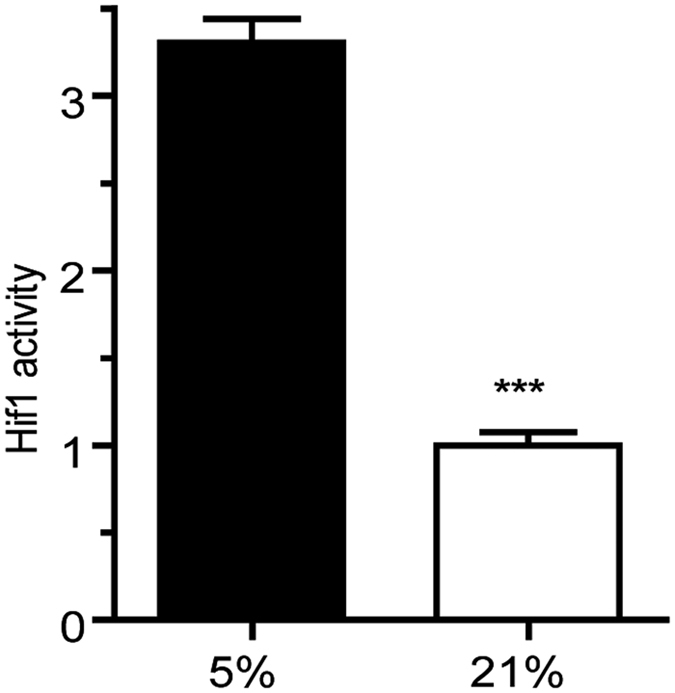
HIF-1α Luciferase assay. Measurement of HIF-1 alpha activity in cells of the OLN93 cell line using a Luciferase assay at 5% O_2_ and 21% O_2_. Statistical analysis revealed a highly significant threefold increase of HIF-1α activity in the 5% O_2_ cultures (p < 0.0001) (N = 4, unpaired ttest p* < 0.05, ** < p0.01, ***p < 0.001).

Many cellular signaling responses to oxygen are mediated by hypoxia-inducible-factor-1 alpha (HIF-1α) activity^14^,^25^. HIF-1α is also known to be a relevant factor for adequate neuronal cell development^26^. In chondrocytes, some specific transcription factors, including Sox9, have been reported to be regulated via HIF-1α activity. Since in our experiments, using oligodendroglial cultures, there was an effect of oxygen levels on the expression of transcription factors Sox9 and Sox10, we aimed to determine the influence of HIF-1α activity on the development of oligodendroglial cells cultured at 5% and at 21% oxygen. For the analysis of HIF-1α activity in cells, reporter gene assay by transient transfection would provide important information. Unfortunately, transfection of plasmid DNA is described to have detrimental effects in primary OPCs, which was also confirmed in our preliminary experiments, with OPCs barely surviving the transfection procedures (data not shown). We therefore used cells of the oligodendroglia cell line OLN93 and subjected them to the two different oxygen levels for 48 hours to determine HIF-1α activity by luciferase expression. Luciferase activity in the OLN93 cultures exposed to 5% was more than 3 times higher than that of the cultures kept at 21% O_2_ (3.30 vs. 1.00, respectively; p < 0.0001) (Fig. 7). Based on these results, the culture condition providing 5% O_2_ increases the activity of Hif-1a by about threefold, and 21% O_2_ may be altering cellular gene expression as a result of decreased Hif-1α activity.

### Gene expression in HIF-1α knockdown cells is mostly decreased compared to wildtype

To clarify whether oligodendroglial development is directly influenced by HIF activity we performed a HIF-1α knockdown in OLN93 cells by utilizing siRNA binding to the HIF-1α mRNA to prevent its expression. To confirm knockdown efficiency, we measured luciferase activity in HIF-1a knockdown cells compared to control siRNA transfected (scrambled) cells incubated at 5% O_2_, indicating 40 pmol as an appropriate siRNA concentration.

**Figure 8:**
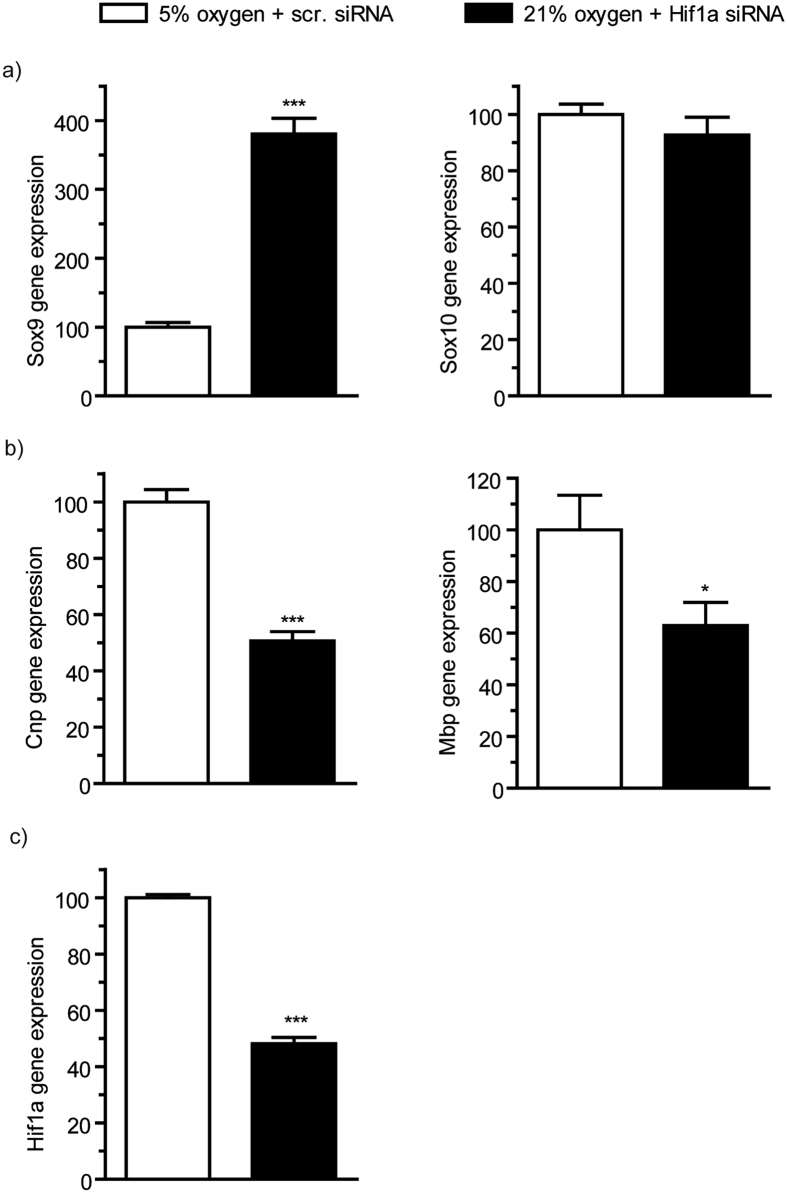
Gene Expression in HIF-1α knockdown. Realtime PCR in HIF-1α knockdown cells of the OLN93 cell line showed a surprisingly strong increase of Sox9 expression (p < 0.0001) and no difference between wildtype and knockdown in Sox10 expression (p = 0.337) (**a**) CNP (p < 0.0001) and MBP (p = 0.044) were significantly downregulated in HIF-1α knockdown cells (**b**) control (**c**) (N = 8, unpaired ttest * < 0.05, ** < p0.01, ***p < 0.001).

We compared real-time PCR analysis of Sox9, Sox10, MBP and CNP in scrambled transected cells cultured at 5% with that in HIF-1α knockdown cells cultured at 5% O_2_, to see whether the upregulation of these genes under the lower oxygen level would be mitigated by the induced HIF-1α deficiency. Surprisingly, Sox9 was significantly upregulated by HIF-1α knockdown (380.25% vs. 100%; p < 0.0001) and Sox 10 showed no significant difference in expression (92.66% vs. 100%; p = 0.337) (Fig. 8a). The expression of MBP and CNP genes, however, was significantly decreased after HIF-1α knockdown cells in comparison to scrambled transfected OLN93 cultured (Fig. 8b, MBP: 62.95% of controls, p = 0.044; CNP: 50.65% of controls; p < 0.0001) indicating a downregulation of maturation signals by HIF-1α at 5% O_2_.

## Discussion

In this study, we investigated the mechanisms through which oligodendroglial development may be regulated by the level of environmental oxygen. In our *in vitro* experiments, low oxygen improved cell survival and morphological complexity compared with 21% oxygen. Moreover, high oxygen decreased cell proliferation and the expression of transcription factors important for the regulation of oligodendroglial development. Both, increased oxidative stress and reduced HIF-1α were identified as potential mediators of the inhibitory effects of higher environmental oxygen.

Oxygen has previously been demonstrated to represent a signal of cell differentiation in stem cells. Chen and co-workers reported that 5% oxygen enhanced clonal and long-term expansion of mouse fetal cortical precursors *in vitro* as compared to 21% oxygen^3^. Low oxygen was shown to promote the development of neural progenitor and stem cells by increasing proliferation and neuronal differentiation under low oxygen^27^,^28^,^29^,^30^,^31^ suggesting a possible role for increased HIF-1α expression^28^,^29^,^30^.

The increased production of ROS and oxidative stress induced by 21% O_2_ may impair OPC development and maturation. Oxidative stress has been shown to impair development and survival in neurons^32^ and oligodendroglia^13^ and has moreover been defined as a major cause of brain injury in neonates^33^,^34^. In our study, we detected elevated nitrotyrosine production in protein samples of oligodendroglial cultures kept at 21% oxygen, indicating oxidative stress. Furthermore, the increased expression of the transcription factor Nrf2 and of SOD2 indicated an anti-oxidant response to oxidative stress. Immature oligodendroglia *in vitro* have been shown to be very susceptible to apoptotic cell death induced by drastically increased oxygen at 80% O_2_^35^. However, the oxidative challenge at 21% O_2_ in comparison to 5% O_2_ does not result in apoptotic activity, as shown by TUNEL analysis in primary OPC cultures. Instead, oxidative stress at 21% oxygen causes changes in central cellular processes, such as proliferation and oligodendroglial differentiation, but does not lead to apoptotic cell death, which is similar to the finding of French *et al*.^13^ and Pistollato *et al*.^36^.

A well-described cellular response to low oxygen concentrations is the upregulation of HIF-1α^14^,^25^,^37^ e.g. in neovascularization^38^ and chondrogenesis^24^,^39^, immune reactions^40^ and different cells of the central nervous system^41^. Recently, oligodendroglial maturation in the immature brain has been found to be strongly regulated by HIF1/2α stabilization and to be reversed by loss of HIF function^42^. It was moreover demonstrated that HIF signaling regulates oligodendroglial development with coupling of angiogenesis and the onset of myelination via paracrine stimulation by oligodendroglia^42^. We hypothesized that changes of oligodendroglial function in response to differences in oxygen concentrations may also be mediated via HIF-1α. Indeed, HIF-1α activity was decreased in our oligodendroglial cultures at 21% O_2_ in comparison to cultures at 5% O_2_. Moreover, in our cell culture experiments using cell line OLN93, at 5% O_2_ environment, HIF-silencing caused a downregulation in MBP and CNP expression to levels similar to 21% oxygen. This data indicates that maturation of oligodendroglia at 5% O_2_ is at least partially regulated by HIF, which represents a novel finding. The regulation of oligodendroglial maturation by HIF should in our view be further investigated.

It should be mentioned that we have also found contradictory results with regards to the expression of Sox9 because Sox9 expression was lower at 21% O_2_ in OPCs and in OLN93 cells but silencing of HIF-1α in OLN93 cells led to upregulation of Sox9. Apparently, in oligodendroglia, Sox9 is not directly regulated via HIF-1α which contrasts with the Hif-1 α dependent cell regulation described in cultured chondrocytes^24^,^43^. Distinct regulatory pathways of HIF-1α and HIF-2 have been reported to provide possible mechanisms of compensation^44^. Given that specific silencing of HIF-1α in our experiments does not fully mimic a broader suppression of HIF activities that is likely to be caused by increased oxygen it is possible that compensatory HIF-2 activity may induce Sox9 up-regulation in the absence of HIF-1.

In our view, the results of our study have technical implications for *in vitro* experiments. Physiological O_2_ levels at which OPCs and oligodendroglia are naturally developing in the cerebral tissue *in vivo* are much lower at about 16–38 mmHg (amounting to 2–5% O_2_)^7^,^45^, oligodendroglial cell cultures maintained at 21% O2, are therefore in fact being exposed to a relatively high O_2_ concentration conditions that interfere with oligodendroglial development. Indeed, we have found that conventional observations of neural cell processes and mechanisms made at 21% O_2_ contain oxygen-mediated growth inhibitory effects. It remains to be determined whether oxygen concentrations of lower than 10% should be used for *in vitro* experiments in OPCs and oligodendroglia to adapt the experimental conditions more closely to the natural situation and avoid oxygen-related impairment. Our results suggest the possibility that lower oxygen levels applied at the beginning of mixed glial culture procedure even before enrichment or purification of OPCs may benefit development.

Our results further indicate that oxidative stress and HIF-related dysregulation of oligodendroglial genes can be avoided by using lower oxygen such as 5% in oligodendroglial cell cultures. This is in agreement with results in neural stem cells, which show improved survival under low oxygen conditions *in vitr*o^46^ and in human mesenchymal stem cells in which proliferation was increased at 2% compared to 20% oxygen^47^. Interestingly, Akundi *et al*. have made similar observations by using OPC cultures at 4% O_2_, however, they defined 4% O_2_ as hypoxia and interpreted their results as an injurious effect of too little oxygen^48^. The possibility has not been previously considered that room air oxygen levels used for *in vitro* experiments might represent supraphysiological oxygen levels for the cellular environment. The degree of vulnerability to the higher oxygen condition will vary between cell types, however, OPCs and immature oligodendroglia are well described to be highly susceptible to oxygen toxicities.

As the primary role of oligodendroglial cells in myelination is the wrapping of oligodendroglial membranes around neuronal axons^49^,^50^ the marked difference in morphological features such as the decreased cell size and substantially less arborization of O4+ cells kept at 21% may lead to myelination deficit. This is a subject for further investigation.

Our results may also provide insights into oxygen-dependent regulation of oligodendroglial development in preterm infants. The intrauterine environment in which the fetus resides is at very low arterial oxygen levels of 20–25 mmHg (or 70–75% oxygen saturation)^2^. Birth into room air exposes the infant to a several-fold increase of oxygen levels, however the preterm brain has poorly developed anti-oxidant defense capacity^10^,^11^,^12^,^51^,^52^,^53^. Cellular differentiation and development in the brain have been shown to be influenced by oxygen^54^. Immature glial cells and progenitor cells are also sensitive to increases of oxygen levels^52^ which can lead to perturbation of the developmental program and to cellular damage. As shown by our data, decreased activity of HIF by higher oxygen concentrations can inhibit transcriptional regulation of oligodendroglial development and maturation.

In conclusion, it is possible that the findings of hypomyelination and diffuse white matter injury in survivors of preterm birth might at least in part be caused by the premature increase in oxygen tension in these patients, leading to oligodendroglial dysregulation by increased oxidative stress and lower HIF activity. The impact of oxygen on the oligodendroglial developmental program should be further investigated in order to understand the causes of white matter injury.

## Methods

### Cell cultures

Primary mixed glial cultures were prepared from E19 pregnant Sprague-Dawley rats by mechanical dissociation according to the method of McCarthy and de Vellis^55^, as previously described^56^,^57^. Mixed cultures (7–10 days old) were shaken for 4 h to detach microglia followed by aspiration of the media to remove the microglia from mixed cultures. After addition of new media, flasks were shaken overnight to detach oligodendrocyte progenitor cells (OPCs) from the astrocyte monolayer. To further minimize contamination by microglial cells, the detached OPC suspension was incubated in succession for 45 min. in 60 mm dishes. OPCs enriched by this method contained >95% GD31 cells labeled by the LB1 monoclonal antibody^58^,^59^, with <0.05% GFAP^+^ astrocytes and <0.05% Ox42^+^ microglia. Mixed glial cultures were used for one shake to produce OPC cultures which were then used for the distinct experiments in a way that N reflects the number of different litters and the number of experiments.

All procedures were approved by the state animal welfare authorities Berlin, Germany, (LAGeSo T-0124/08) and followed institutional guidelines.

### OLN93 cells

We received cells of the oligodendroglia linage cell line OLN93^23^ from Dr. C. Richter-Landsberg (Oldenburg, Germany). Cells were cultured as described before (Gerstner *et al*.,)^35^,^60^. Briefly, for immunocytochemistry, cells were transferred on poly-lysine-coated coverslips kept in 12-well plates at a density of 1.5 × 10^5^ cells per well and incubated at 5% and 21% oxygen for 48 hours.

For Hif-1α gene reporter assay 0.5 × 10^5^ cells per 24-well were transfected with the commercial available HIF-1α reporter assay (Qiagen) by lipofection (Metafectene Pro, Biontex) according to the manufactures guide lines and incubated at 5% and 21% O_2_. After 48 hours incubation, cells were harvested and preceded with the Dual-Luciferase Reporter Assay System (Promega). Luminescence was measured by the Lumat LB 9501 Luminometer (Berthold).

For gene expression analyses, 1.5 × 10^5^ cells per 12-well were incubated at 5% and 21% oxygen. After 48 hours incubation cells were preceded for Real-time PCR analyses.

Gene silencing by Hif-1α siRNA (Santa Cruz Biotechnology) was performed in 0.5 × 10^5^ cells per 24-well. siRNA transfection and co-transfection with the Hif-1α reporter assay was performed by lipofection (Metafectene Pro, Biontex) according to the manufactures guide lines and incubated at 5% and 21% O_2_ for 48 hours.

### Western blot

Whole cells lysates were homogenized in 4 °C RIPA buffer solution for protein extraction. Protein concentration was measured by the Pierce BCA kit (Pierce, Rockford IL). Total proteins were equally loaded using 20 μg per lane on a 12% mini precast Tris-glycine gel. Proteins were transferred onto PVDF membranes at 4 °C overnight and blocked in 3% BSA in TBST. Primary antibodies were diluted 1:1,000 to 1:1,250 in 3% BSA/TBST. Horseradish peroxidase (HRP)-conjugated secondary antibodies (anti-rabbit and anti-mouse, BD Biosciences Pharmingen, San Diego CA) were diluted 1:2,000 in 3% BSA/TBST. Chemiluminescent detection was performed using the ECL Plus (Amersham) kit according to manufacturers’ directions. Positive signals were visualized using enhanced chemiluminescence (Amersham Biosciences, Freiburg, Germany) and quantified using a ChemiDoc XRSþ system and the software Image Lab (Bio-Rad). The antibodies used were monoclonal mouse anti-β-actin 1:1,250 (Millipore/Chemicon, Temecula CA), polyclonal rabbit anti-nitrotyrosine 1:1,000 (Millipore/Upstate, Temecula CA).

### Immunocytochemistry

Live staining for cell surface antigens with A2B5 and O4 antibodies^61^ was performed as described elsewhere^62^. Briefly, live cells were incubated at room temperature for 1 hour with primary antibodies diluted 1:10 in DMEM, followed by fluorescein-conjugated goat anti-mouse IgM for 45 min. After three washes in phosphate-buffered saline solution (PBS), cells were fixed in 4% paraformaldehyde (PFA) in PBS for 10 min. at room temperature and washed in PBS. Coverslips were then mounted in DAPI (4′,6-diamidino-2-phenylindole)-Containing Vectashield. For double staining with Ki67, cells after live staining, fixation and washing were blocked again in 10% normal goat serum (NGS) in DMEM containing 0.1% Triton X-100 for permeabilization during 20 min. at room temperature. Incubation with Ki67 rabbit antibody (1:500, Dako) followed for 1 hour at room temperature. After washing, the cells were incubated with rhodamine anti-rabbit IgG antibodies (1:200, JacksonImmuno, West Grove PA). The cells were then washed and mounted in Vectashield with DAPI. For TUNEL staining, a commercially available kit (RocheDiagnostics, Mannheim, Germany) was used according to the manufacturer’s instructions after live-staining, fixation and washing of the cells.

OLN93 cells were fixed with 4% PFA in PBS for 15 minutes. After washing with PBS, cells were permeabilized with 0.1% Triton X 100 and 0.1% Tween 20 in PBS for 10 minutes. After three washing steps, the cells were blocked with 0.5% BSA in PBS for 1 hour at room temperature, followed by the incubation with the rabbit anti Ki67 (Leica, NCL-Ki67p) 1:200 in 0.5% BSA in PBS for one hour. After several washing steps, the cells were incubated with a goat-anti-rabbit IgG Alexa Fluor 594 (Molecular Probes) 1:200 for 30 minutes. The cells were then washed and mounted in Vectashield with DAPI.

Images were taken at 20 fold magnification. Five images were taken per 24 mm cover slip covering north/south/east/west/center regions of the each cover slip. The average of the five images was used as n = 1.

### Sholl analysis

After live staining for cell surface antigens with O4 was conducted, evaluation of cell size and morphology was mastered using Sholl Analysis, an ImageJ plugin used to describe neuronal arbors^63^. Images were first converted into binary 8-bit images and the center of each cell was marked using the ImageJ point selection tool. Sholl analysis was then conducted scaled to pixels, the minimum radius being 0.00 and the radius step being set to 0.032 pixels.

### Real-time PCR

Total cellular RNA was isolated by acidic phenol/chloroform extraction and DNase I treatment (Qiagen, Hilden Germany). Prior to reverse transcription, DNA contamination was ruled out by running RNA samples on a 2% agarose gel subsequently developed with ethidium bromide. For each sample, 1 μg of RNA was reverse transcribed at 42 °C for 1 h with 200 U of Moloney murine leukemia virus reverse transcriptase and random-primer (Promega, Madison, WI) in 35 μl of reaction mixture including DNaseI treatment. For qPCR analysis, 3 μL of 1:10 diluted cDNA samples were used. The PCR products of *Sox-9, Sox-10, Olig1, Olig2*, 2′3′ cyclic nucleotide phosphodiesterase *(CNP)*, myelin basic protein *(MBP), superoxide dismutase 2 (SOD2) and NF-E2-like basic leucine zipper transcriptional activator(NRF2)* were quantified in real-time, using dye-labeled fluorogenic reporter oligonucleotide probes (*Sox-9* F 5′cggaggaagtcggtgaagaa 3′, R 5′tgcagcgccttgaagatg 3′, probe 5′acagactcacatctctccta3 3′, NM_001109181.1, *Sox-10* F 5′ ccgcacctccacaatgct 3′, R 5′ ggtacttgtagtccggatggtcttt 3′, probe 5′ ttgctgaacgagagtgacaa 3′, NM_019193.1; *Olig1* F 5′ ccctcgcgtcctggatct 3′, R 5′ ggaagaaggcgccctacag 3′, probe 5′ aaaggaggacatttccagac 3′, NM_021770;*Olig2* F 5′ tgcgcaagctctccaagat 3′, R 5′ tctcgctcaccagtctcttcatc 3′, probe 5′ cgaaactacatcctgatgct 3′, NM_001100557.1;*CNP* F 5′ ggcgtgctgcactgtacaac 3′, R 5′ aagatctcctcaccacatcctgtt 3′, probe 5′ aattctgtgactacgggaag 3′, NM_012809.1; *MBP* F 5′ gagccctctgccttctcatg 3′, R 5′ agggagccgtagtgggtagttc 3′, probe 5′ acatgtacaaggactcacac 3′, NM_001025291.1, *SOD2* F 5′gacctacgtgaacaatctgaacgt 3′, R 5′ aggctgaagagcaacctgagtt 3′, probe 5′accgaggagaagtaccacga 3′, NM_017051.2, *NRF2* F5′actcccaggttgcccacat 3′, R 5′gcgactcatggtcatctacaaatg 3′, probe F 5′ctttgaagactgtatgcagc 3′, NM_017051.2).

The probes were labeled at the 5′ end with the reporter dye 6-carboxy-fluoresceine (FAM) and at their 3′ ends with the quencher dye, 6-carboxy-tetramethylrhodamine (TAMRA).Hypoxanthine-guanine phosphoribosyl-transferase (*HPRT,* F 5′ ggaaagaacgtcttgattgttgaa 3′, R 5′ccaacacttcgagaggtcctttt 3′, probe 5′ ctttccttggtcaagcagtacagcccc 3′, NM_013556.2) was used as internal standard. The FAM spectral data was collected from reactions carried out in separate tubes using the same stock of cDNA to avoid spectral overlap among FAM/TAMRA and limitations of reagents. Real-time PCR was performed in three replicates and repeated two times of each sample using a total reactive volume of 11 μl which contained 5 μl of 2x KAPA PROBE FAST qPCR Mastermix (PEQLAB Biotechnologie GMBH, Erlangen, Germany), 2.5 μl of 2 μM oligonucleotide mix (forward and reverse primer, BioTeZ Berlin-Buch, Germany), 0.5 μM probe, and 9 ng of cDNA template (diluted in RNase-free water to 3 μl). The PCR amplification was performed in 96-well optical reaction plates for 40 cycles with each cycle at 94 °C for 15 sec and 60 °C for 1 min. Each plate included at least three “No Template Controls (NTC)”. The reaction was carried out with the StepOnePlus™ Real-Time PCR System (Applied Biosystems) according to the 2^−^ddCT method, and fluorescent data were converted into cycle threshold (C_T_) values. *Sox-9, Sox-10, Olig1, Olig2, CNP, MBP, SOD2, NRF2* levels were normalized to *HPRT* levels. Results were normalized in per cent with the 5% oxygen culture group used as control group.

### Statistical analysis

All of the results were analyzed by student’s t-test, RT-PCR was analyzed using Wilcoxon signed rank test, *P* < 0.05 was defined as statistically significant. All values are given as mean ± standard error of the mean (SEM).

## Additional Information

**How to cite this article:** Brill, C. *et al*. Oxygen impairs oligodendroglial development via oxidative stress and reduced expression of HIF-1α. *Sci. Rep.* **7**, 43000; doi: 10.1038/srep43000 (2017).

**Publisher's note:** Springer Nature remains neutral with regard to jurisdictional claims in published maps and institutional affiliations.
