# Corrigendum: Oxygen impairs oligodendroglial development via oxidative stress and reduced expression of HIF-1α

**DOI:** 10.1038/srep46800

**Published:** 2017-05-12

**Authors:** Christina Brill, Till Scheuer, Christoph Bührer, Stefanie Endesfelder, Thomas Schmitz

Scientific Reports 7: Article number: 4300010.1038/srep43000; published online: 02 23 2017; updated: 05 12 2017

This Article contains an error in Figure 8: the oxygen percentage for the black columns is incorrectly labeled as 21% instead of the correct value of 5%. The correct Figure 8 appears below as Figure 1 along with the accompanying legend.

## Figures and Tables

**Figure 1:**
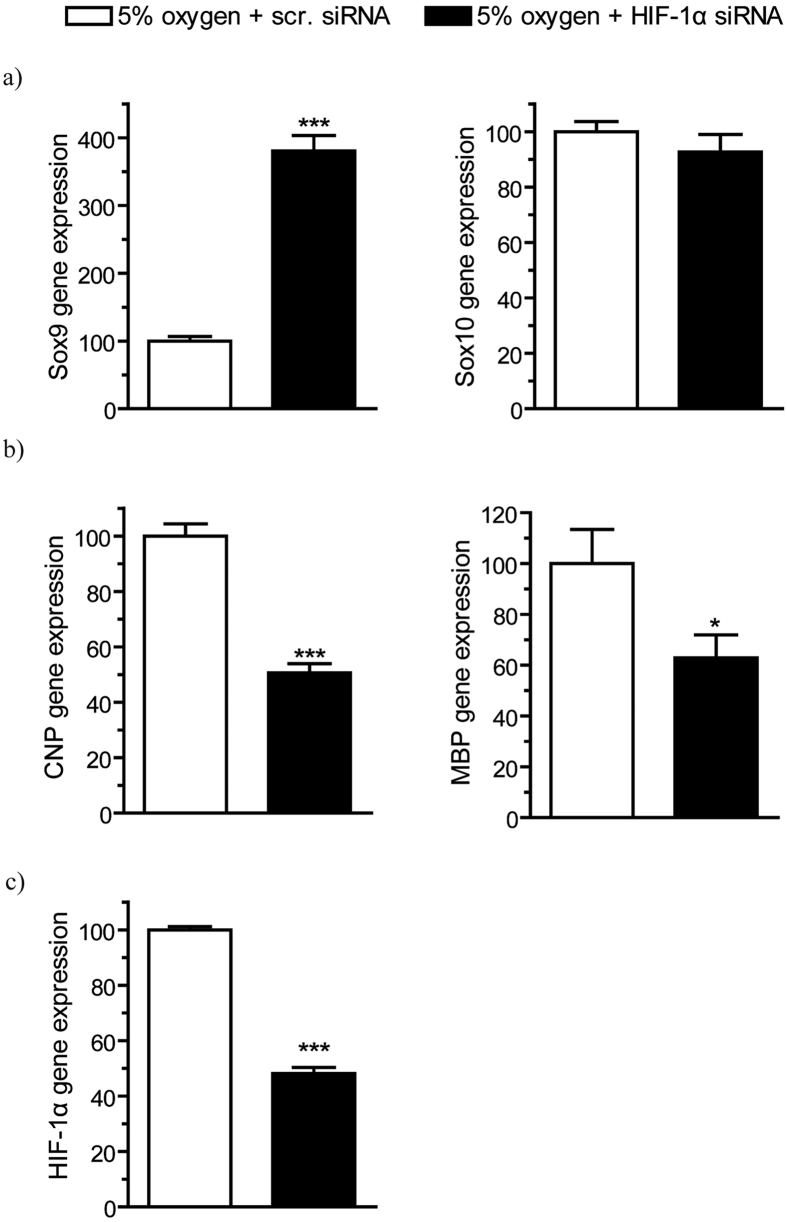
Realtime PCR in HIF-1α knockdown cells of the OLN93 cell line showed a surprisingly strong increase of Sox9 expression (p < 0.0001) and no difference between wildtype and knockdown in Sox10 expression (p = 0.337), as shown in (a). CNP (p < 0.0001) and MBP (p = 0.044) were significantly downregulated in HIF-1α knockdown cells as compared to control with scrambled siRNA, which can be seen in (b). Successful knockdown of HIF-1α by specific siRNA is demonstrated in (c). (N = 8, unpaired t test *p < 0.05, **p < 0.01, ***p < 0.001)

